# Structural and functional assessment of TBX20 gene variants in pediatric ventricular septal defect

**DOI:** 10.1186/s41065-025-00513-5

**Published:** 2025-08-04

**Authors:** Zhenzhen Qin, Caixia Liu, Jie Wang, Yanmei Jin

**Affiliations:** 1 Department of Cardiothoracic Surgery, Children’s Hospital of Shanxi, No. 65 of Jinxi Street, Jinyuan District, Taiyuan, 030025 Shanxi Province China; 2 Department of Burn and Orthopedics, Children’s Hospital of Shanxi, Taiyuan, 030025 Shanxi Province China; 3 Department of General Surgery, Meizhou People’s Hospital, Meizhou, 514031 Guangdong Province China

**Keywords:** Gene variants, Pediatric population, T-box transcription factor, TBX20, Ventricular septal defect

## Abstract

**Objective:**

This study aimed to investigate the potential role of TBX20 gene variants in the molecular pathogenesis of congenital ventricular septal defect (VSD) in pediatric patients.

**Methods:**

Genetic sequencing and variant detection were performed for the TBX20 gene, a T-box transcription factor, in a cohort of 150 pediatric patients diagnosed with VSD, recruited from the Department of Cardiothoracic Surgery at Shanxi Children's Hospital. Functional characterization of newly identified variants was conducted using homology-based protein structural modeling, dual-luciferase reporter assays, and quantitative real-time polymerase chain reaction (qRT-PCR).

**Results:**

Two variants within the highly conserved T-box DNA-binding domain were identified in five children: a synonymous variant c.576C > T (p.Thr192Thr) and a missense variant c.577G > A (p.Gly193Ser). Structural modeling predicted that the p.Gly193Ser substitution destabilized the TBX20 protein by altering its conformation and increasing its potential energy state. Functional assays demonstrated that this variant reduced TBX20 mRNA expression and significantly attenuated transactivation of the downstream target gene *ANF*. Bioinformatic analysis supported the deleterious functional impact of the p.Gly193Ser variant and its potential contribution to VSD pathogenesis. In contrast, the synonymous p.Thr192Thr variant was associated with increased transcriptional activity of TBX20 and enhanced regulation of *ANF*. qRT-PCR data indicated significantly reduced TBX20-G193S mRNA levels compared to wild-type (WT) when expressed independently (*p* < 0.01), but elevated levels in the presence of GATA4 and NKX2-5 (*p* < 0.001). Despite this, ANF transactivation remained significantly lower than WT, suggesting impaired functional capacity. These alterations may influence translational efficiency and contribute to abnormal cardiac septation.

**Conclusion:**

The findings underscore the involvement of TBX20 gene variants in the etiology of pediatric VSD and provide mechanistic insights that may inform future clinical research and the development of targeted therapeutic strategies.

**Supplementary Information:**

The online version contains supplementary material available at 10.1186/s41065-025-00513-5.

## Introduction

Congenital heart disease (CHD) represents the most common congenital anomaly among neonates, encompassing a range of cardiovascular malformations arising from disruptions in cardiac and vascular development during embryogenesis [1]. The prevalence of CHD in live-born neonates is estimated to range from 0.6% to 1% [2]. CHD significantly impacts growth, neurodevelopmental outcomes, and quality of life in affected children, and remains a leading cause of neonatal and infant mortality and long-term disability. The etiology of CHD is multifactorial and incompletely understood, involving a complex interplay of genetic predisposition, environmental exposures, and potential teratogenic factors, including maternal infections during pregnancy. These influences may give rise to chromosomal abnormalities or gene variants affecting key regulatory pathways in cardiac morphogenesis [3, 4].

Although advancements in diagnostic and therapeutic strategies have substantially improved outcomes for many patients with CHD, those with complex cardiac anomalies often require multiple surgical interventions or cardiac transplantation. These interventions impose significant financial and psychological burdens on affected families and healthcare systems [5, 6]. Therefore, identification of susceptibility or causative genes and elucidation of the molecular mechanisms underlying CHD are essential for informing targeted prevention and early intervention strategies.

The T-box family of transcription factors is known to play a critical role in various stages of cardiogenesis. Among them, TBX20 has been identified as a key regulator of myocardial development and has been implicated in the pathogenesis of several forms of structural heart disease [7, 8]. TBX20 protein is expressed in multiple cardiac cell types, where it coordinates with other transcription factors, including NKX2-5 and GATA4. These interactions facilitate the transcriptional activation of downstream targets such as *CX40*, *CX45*, and *ANF*, the latter of which has been extensively studied [9, 10]

Experimental studies in zebrafish have shown that loss-of-function variants in the TBX20 gene lead to a marked reduction in cardiac progenitor cells, resulting in a hypoplastic, nonfunctional heart. In contrast, overexpression of TBX20 increases cardiomyocyte numbers and leads to cardiac enlargement. This effect is attributed to enhanced formation of cardiac progenitor cells and increased cardiomyocyte proliferation, both dependent on the activity of the TBX20 protein's transcriptional activation domain [8].

In humans, TBX20 gene variants have been associated with several congenital heart malformations, including atrial septal defect, double outlet right ventricle, tetralogy of Fallot, and ventricular septal defect (VSD) [11–13]. VSD is the most common type of congenital heart defect, and growing evidence supports a significant genetic component in its etiology [14].

To investigate the pathogenic potential of TBX20 gene variants in children diagnosed with VSD, targeted sequencing of exons 2 through 6 of the TBX20 gene was performed using peripheral venous blood samples obtained from a cohort of 150 affected children. Newly identified synonymous and missense variants located within the conserved T-box domain were subsequently subjected to functional analysis to evaluate their structural and transcriptional effects.

## Materials and methods

### Study participants

The case group consisted of 150 children (83 males and 67 females) diagnosed with congenital VSD. Participants were consecutively recruited between January 2015 and March 2017 from the Department of Cardiothoracic Surgery at Shanxi Children's Hospital. Diagnosis of VSD was established through a combination of physical examination, transthoracic echocardiography, and intraoperative findings. Peripheral venous blood samples were collected from all children in the case group, and leukocytes were isolated for genomic DNA extraction.

A control group of 150 healthy children (72 males and 78 females), who underwent routine physical examinations at the outpatient department of Shanxi Children's Hospital during the same period, was also included. All control participants had normal findings on cardiac color Doppler ultrasound and no known history of congenital or acquired cardiac abnormalities.

Written informed consent was obtained from the legal guardians of all participants in both the case and control groups prior to enrollment in the study.

## Research methods

### Gene sequencing

Genomic DNA was extracted from peripheral venous blood samples obtained from children in both the case and control groups, using the Biogenic Genomic DNA Extraction Kit, following the manufacturer’s protocol. Exons 2 to 6 of the TBX20 gene, which encode the T-box domain, were amplified by polymerase chain reaction (PCR) using specifically designed upstream and downstream primers:

Exon 2: CCCAGGTAATTTTATACAGTGAT (23 bp) CTAGACATCCTGTAGCTCCTAAT (23 bp).

Exon 3: GAGTCAGACCCTTTCCCTCC (20 bp) AGGCTTGGAATGCTCTCTTG (20 bp).

Exon 4: CCCACTTATATATGGTTTATGTGTTCC (27 bp) AGATAGAAGGTGGGAAGGGG (20 bp).

Exon 5: TTAAGGCTGGAAAGGGTAGAT (21 bp) AGGTGTGGGTGGGGAAGTG (19 bp).

Exon 6: TTCCACCCTTCTCAGGACAC (20 bp) AGGCCTGCCTGATGTCTCT (19 bp).

PCR amplification was carried out in a 50 μL reaction volume containing 50 ng of genomic DNA, 1 × PCR Buffer (Mg^2^⁺ Plus), 0.15 mM dNTPs, 0.3 μM of each primer, and 2 U of Taq DNA polymerase (TaKaRa, Dalian Bao Biological Co.). Amplification was performed using a PTC-200 thermal cycler (USA).

Following amplification, PCR products were purified and sequenced using an ABI 3700 DNA sequencer. The resulting sequences were aligned against the reference human genome using the BLAST tool on the National Center for Biotechnology Information (NCBI) website. Sequence chromatograms were also analyzed using Chromas software and compared to wild-type sequences from NCBI to identify variants. All sequence data were additionally compared with the GenBank reference for the human *TBX20* gene (Gene ID: 57,057) to detect novel variants.

## Variant pathogenicity prediction analysis

To evaluate the potential pathogenicity of identified synonymous and missense variants, computational prediction tools were employed. For the synonymous variant c.576C > T (p.Thr192Thr), pathogenicity prediction was performed using the integrated modeling software available at http://www.xialab.info:8080/PrDSM/. Because this tool requires input based on the hg19 reference genome, the initial genomic coordinates based on the NCBI hg38 reference genome were converted accordingly prior to analysis. The software was then used to assess whether the T192T variant is likely to represent a deleterious synonymous variant.

The TBX20 protein sequence was initially retrieved from the UniProt database. Pathogenicity prediction for the TBX20-G193S variant was subsequently performed using bioinformatics tools including PolyPhen-2 (http://genetics.bwh.harvard.edu/pph2/), MutationTaster (http://www.mutationtaster.org/), and the Ensembl genome browser (http://grch37.ensembl.org/Homo_sapiens/Info/Index). The following online tools were used to predict the pathogenicity of missense variants:

- **PolyPhen-2:** http://genetics.bwh.harvard.edu/pph2/
- **MutationTaster:** http://www.mutationtaster.org/
- **SIFT:** http://blocks.fhcrc.org/sift/SIFT.html
- **PMut:** http://mmb2.pcb.ub.es:8080/PMut/

## Homology modelling of TBX20 protein structure

Homology modelling, also referred to as comparative modelling, was used to predict the three-dimensional (3D) structure of the TBX20 protein based on a homologous protein with a known structure. The amino acid sequence of TBX20 was retrieved from the UniProt protein database. TBX1 (Protein Data Bank ID: 4A04) was selected as the template for model construction. Single-template modelling was performed using Modeller version 9.20 to generate 3D structures of both the wild-type TBX20 (TBX20 WT) and the variant form TBX20-G193S [15, 16]. Additionally, the I-TASSER protein structure prediction server was used to construct comparative models. Structural analysis was subsequently conducted for both the wild-type and variant TBX20 protein models.

## Assessment of TBX20 mRNA expression levels by qRT-PCR

The pcDNA3.1-TBX20 wild-type (WT) expression plasmid was constructed (Shanghai Sangong Biotechnology Co., Ltd., China). Site-directed mutagenesis was used to introduce the synonymous variant T192T and the variant TBX20-G193S into the plasmid. COS-7 cells were transfected with the pcDNA3.1-TBX20 WT, -T192T, and -G193S expression plasmids, along with an empty pcDNA3.1 plasmid serving as a blank control.

For transfection, 2 µg of plasmid DNA and 4 µL of liposome transfection reagent were each diluted in Opti-MEM medium, combined, and incubated at room temperature for 20 min to form DNA–liposome complexes. These complexes were then added to COS-7 cells. After incubation at 37 °C for 6 h, the transfection medium was replaced with complete culture medium, and cells were incubated for an additional 48 h.

The experimental groups were defined as follows:

Group A consisted of cells transfected with 2 µg of empty pcDNA3.1 plasmid and 4 µL of transfection reagent, serving as the blank control. Group B received 2 µg of the TBX20 WT expression plasmid and 4 µL of transfection reagent. Group C was transfected with 2 µg of the TBX20-G193S variant plasmid and 4 µL of transfection reagent, while Group D received 2 µg of the TBX20-T192T variant plasmid and 4 µL of transfection reagent. In Group E, cells were co-transfected with 1 µg of TBX20 WT plasmid, 0.5 µg TK-RLuc TK-RLuc of pcDNA3.1-*GATA4* plasmid, and 0.5 µg of pcDNA3.1-*NKX2-5* plasmid, along with 4 µL of transfection reagent. Group F included 1 µg of TBX20-G193S plasmid with 0.5 µg each of GATA4 and NKX2-5 plasmids and 4 µL of transfection reagent. Group G received 1 µg of TBX20-T192T plasmid, 0.5 µg of GATA4 plasmid, and 0.5 µg of NKX2-5 plasmid, also with 4 µL of transfection reagent.

Total RNA was extracted from cells 48 h post-transfection using TRIzol reagent. Specifically, 1 mL of TRIzol was added per 5 × 10⁶ cells, followed by pipette homogenization to ensure complete cell lysis. The lysate was transferred to a 1.5 mL microcentrifuge tube, and 200 µL of chloroform was added per mL of TRIzol. The mixture was inverted for 15 s, incubated at room temperature for 10 min, and centrifuged at 12,000 rpm for 15 min at 4 °C. The upper aqueous phase was transferred to a new tube and mixed with an equal volume of cold isopropanol. Following incubation at 4 °C for 10 min, RNA was precipitated by centrifugation at 12,000 rpm for 10 min at 4 °C. The RNA pellet was washed twice with 75% ethanol (prepared in DEPC-treated water), centrifuged at 10,000 rpm for 5 min at 4 °C, and air-dried at room temperature.

Quantitative real-time PCR (qRT-PCR) was performed using SYBR Green chemistry to quantify TBX20 mRNA expression. Each 20 µL reaction mixture contained the following components: 10 µL SYBR Green PCR Master Mix, 0.5 µL of 2.5 µM forward primer, 0.5 µL of 2.5 µM reverse primer, 1.0 µL of template cDNA, 0.4 µL ROX reference dye, and 7.6 µL of deionized water.

Two primer pairs were used for amplification:

- Primer pair 1 (PCR product d1):
- Forward (Primer 1): GGAGACCCAAGCTGGCTAGC
- Reverse (Primer 2): AGTTGCTCACTGGTAAAAGGAGAATC
- Note: Primer 2 includes the G193S variant site.
- Primer pair 2 (PCR product d2):
- Forward (Primer 3): TCCTTTTACCAGTGAGCAACTACTCA
- Reverse (Primer 4): GGTTTAAACGGGCCCTCTAGA
- Note: Primer 3 includes the G193S variant site.

The thermal cycling protocol was as follows:

- Initial denaturation: 95 °C for 30 s
- Amplification (40 cycles): 95 °C for 5 s, 60 °C for 34 s (fluorescence acquisition during extension)

Melting curve analysis was conducted immediately after amplification to confirm product specificity, using the following settings:

- 95 °C for 1 min (denaturation)
- 55 °C for 1 min (cooling)
- Temperature ramp from 55 °C to 95 °C in 0.5 °C increments over 81 cycles, with a 4-s hold and fluorescence acquisition at each step

Additional co-transfection experiments were performed in COS-7 cells using expression plasmids for NKX2-5 and GATA4 in combination with TBX20 WT, T192T, G193S, or the empty pcDNA3.1 vector. The same qRT-PCR protocol, as described above, was used to assess TBX20 mRNA expression under the combined regulatory influence of NKX2-5 and GATA4.

Relative expression levels were quantified using the 2^–ΔΔCt method. Statistical comparisons among groups were conducted using one-way analysis of variance (ANOVA), and data analysis was performed with SPSS software, version 24.0.

## Effect of TBX20 gene variants on ANF promoter activity assessed by dual-luciferase reporter assay

The ANF gene is a well-characterized downstream target of TBX20. The T-half site, a specific DNA element located in the ANF promoter region, is capable of binding to the T-box domain of TBX20. Transcriptional activation of the ANF promoter is further enhanced by the synergistic activity of GATA4 and NKX2-5.

To assess the regulatory effect of TBX20 variants on ANF promoter activity, a dual-luciferase reporter assay was performed. A 2,600 bp fragment of the ANF promoter region was cloned into the pGL3-Basic luciferase reporter vector to generate the pGL3-ANFluc construct (Biotechnology, Shanghai, China). Expression plasmids encoding pcDNA3.1-TBX20 WT, pcDNA3.1-TBX20 T192T, pcDNA3.1-TBX20 G193S, pcDNA3.1-GATA4, and pcDNA3.1-NKX2-5 were also constructed.

COS-7 cells were transfected in four groups using combinations of the *TBX20* constructs (WT, T192T, G193S, or empty pcDNA3.1), along with the pGL3-ANFluc plasmid, the Renilla luciferase control plasmid TK-Rluc (Renilla luciferase,TK-RLuc), and expression plasmids for GATA4 and NKX2-5. After 48 h of transfection, cells were harvested, and luciferase activity was measured using a dual-luciferase reporter assay system according to the manufacturer’s instructions.

The transfection mixtures for each group were prepared as follows:

- Group A: 190 ng pcDNA3.1, 300 ng pGL3-ANFluc plasmid, 10 ng TK-Rluc plasmid, and 1 µL transfection reagent
- Group B: 90 ng TBX20-WT plasmid, 50 ng NKX2-5 plasmid, 50 ng GATA4 plasmid, 300 ng pGL3-ANFluc plasmid, 10 ng TK-Rluc plasmid, and 1 µL transfection reagent
- Group C: 90 ng TBX20-G193S plasmid, 50 ng NKX2-5 plasmid, 50 ng GATA4 plasmid, 300 ng pGL3-ANFluc plasmid, 10 ng TK-Rluc plasmid, and 1 µL transfection reagent
- Group D: 90 ng TBX20-T192T plasmid, 50 ng NKX2-5 plasmid, 50 ng GATA4 plasmid, 300 ng pGL3-ANFluc plasmid, 10 ng TK-Rluc plasmid, and 1 µL transfection reagent

Each DNA–liposome complex was diluted in 100 µL of serum-free, antibiotic-free DMEM, mixed thoroughly, and incubated at room temperature for 20 min before being applied to the cells. All transfections were performed in triplicate. Luciferase activity was normalized to Renilla luciferase activity, and results were expressed as mean ± standard deviation.

## Statistical analysis

Statistical analyses were performed using SPSS software, version 24.0 (IBM Corporation, Armonk, NY, USA). Relative mRNA expression levels obtained from qRT-PCR were calculated using the 2^–ΔΔCt method. Results from the dual-luciferase reporter assay were expressed as relative fluorescence values, calculated as the ratio of firefly luciferase to Renilla luciferase activity. All experiments were conducted in triplicate, and data were presented as mean ± standard deviation. Differences among multiple groups were evaluated using one-way analysis of variance (ANOVA). A *p*-value of < 0.05 was considered statistically significant.

## Results

### Identification of *TBX20* variants

Two TBX20 gene variants—a synonymous variant (T192T) and a missense variant (G193S)—were identified in five children diagnosed with VSD (Supplementary Fig. 1). These variants were not detected in any participants from the control group. The synonymous variant (c.576C > T) involves a substitution of cytosine with thymidine and does not alter the encoded amino acid sequence. The missense variant (c.577G > A) results in an amino acid substitution from glycine (Gly) to serine (Ser), corresponding to the G193S variant.

Conservation analysis using the NCBI HomoloGene database demonstrated that the affected amino acid residues are highly conserved across multiple species, including humans and other vertebrates, indicating potential functional significance (Supplementary Fig. 2).

## Pathogenicity prediction of TBX20 variants

### G193S missense variant

The pathogenic potential of the G193S missense variant was evaluated using multiple bioinformatics tools [17], including PolyPhen-2 [18], MutationTaster [19], Sorting Intolerant From Tolerant (SIFT) [20], REVEL (https://sites.google.com/site/revelgenomics/downloads) and MetalR (https://github.com/Schuture/MetaLR) [21] as required (Supplementary Fig. 3). All prediction platforms indicated that the variant is likely pathogenic.

The pathogenicity scores were as follows:

- PolyPhen-2: 0.98 (score approaching 1.0 indicates a high probability of being damaging)
- REVEL: 0.916
- MetaLR: 0.981

MutationTaster predicted the variant to be disease-causing, with potential effects on both protein structure and function. The SIFT score for G193S was 0.00, indicating high confidence in its deleterious effect (scores < 0.05 are considered damaging). Together, these results suggest that the G193S variant is likely to impair TBX20 protein function and may contribute to disease pathogenesis.

## T192T synonymous variant

The synonymous variant T192T was analyzed using the Prediction of Deleterious Synonymous Mutation (PrDSM) tool, an integrated software model designed to assess the functional consequences of synonymous variants [17]. As the NCBI database uses the hg38 reference genome and the PrDSM tool is based on hg19, genome coordinate conversion was required prior to analysis. Genomic localization was performed using the NCBI genome browser, with sequence data exported in text format to identify the variant site. The Integrative Genomics Viewer (IGV) was then used to determine the corresponding position on the hg19 assembly.

As TBX20 is located on the negative strand, the reverse complementary sequence of the reference genome was used for accurate mapping of the variant site. The results obtained from the PrDSM analysis suggest that the T192T variant is most likely benign and does not significantly affect gene expression or protein translation (Table 1).

**Table 1:** Pathogenicity prediction of synonymous mutation (T192T)

Gene Name	Chr	Pos	GeneID	TranscriptID	Re f	Alt	TraP	SilVA	FATHMM-MKL	PrDSM
TBX20	7	35,284,639	ENSG00000164532	ENST00000408931	G	A	0.003996004	0.044071354	0.084179158	0.044082172

TraP: Score <0.005, indicating that pathogenic may be <10%; score <0.015, indicating that pathogenic may be 10% -25%;

SilVA: Score 0~1; (> 0.278 indicates that it may be pathogenic, the closer to 1, the higher the possibility of pathogenic);

FATHMM-MKL: Score 0~1 (> 0.5 indicates that pathogenic and higher score may be pathogenic);

PrDSM: Score 0~1 (> 0.308 indicates the presence of pathogenic).

## Structural modeling of TBX20 protein and conformational changes associated with the G193S variant

### TBX 20 protein structure prediction

**Fig. 1:**
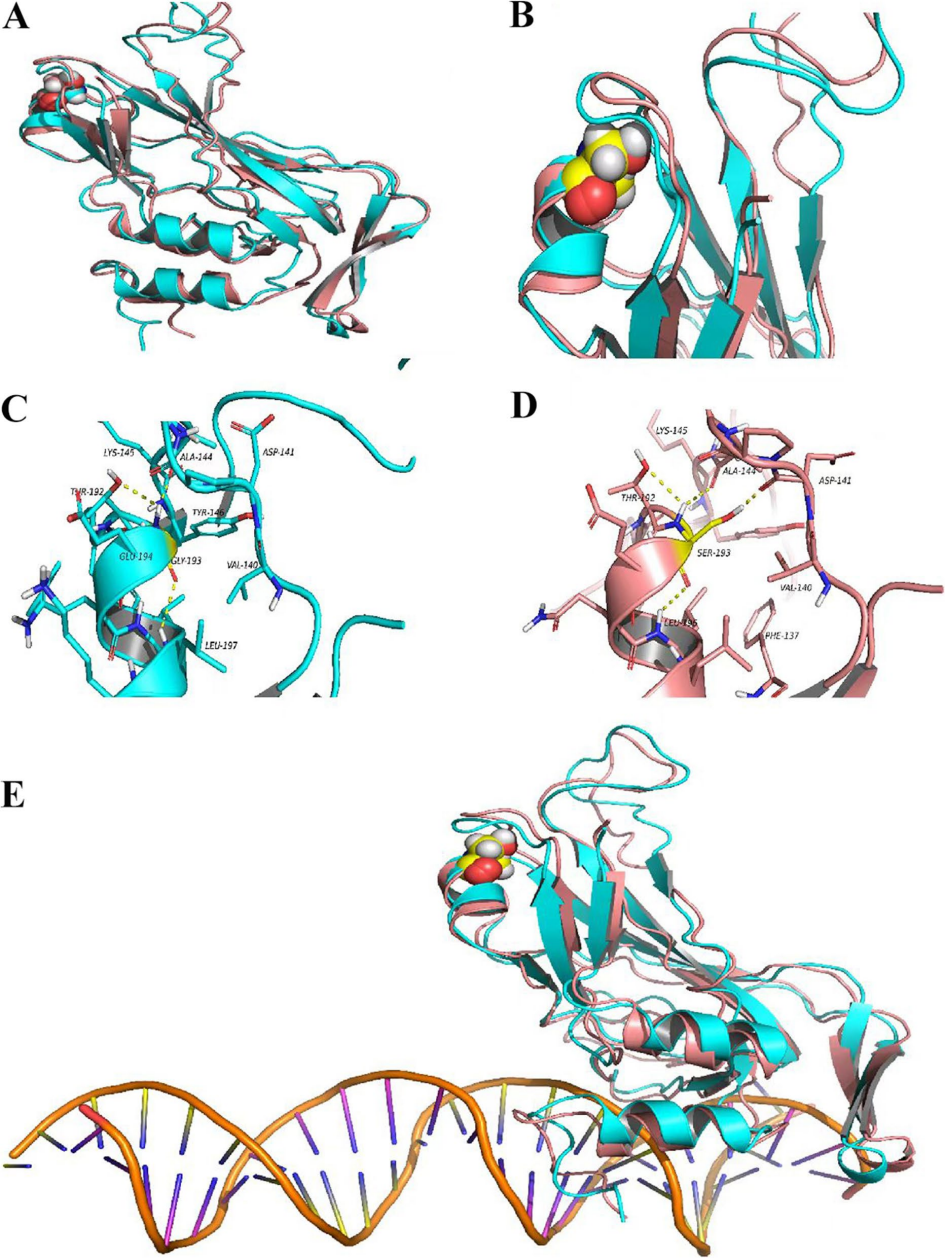
Homology modeling of TBX20 wild-type and G193S variant protein structures. **A:** Predicted structures of wild-type TBX20 and the G193S variant. **B**: The loop region adjacent to Ser193 in the G193S variant shifts outward, altering the conformation of the surrounding loop. Note: Light blue ribbon represents wild-type TBX20; orange ribbon represents TBX20-G193S. Key amino acid residues are shown as spheres and colored by atom type: blue (nitrogen), white (hydrogen), red (oxygen), yellow (carbon). **C**: In wild-type TBX20, Gly193 is adjacent to Ala144, Thr192, and Leu197 and forms hydrogen bonds. **D**: In the TBX20-G193S variant, Ser193 is adjacent to Asp141, Ala144, Thr192, and Leu196, with altered hydrogen bonding patterns. Note: Affected residues are labeled with black letters and numbers. Stick structures indicate nearby amino acids, colored by atom type as above. Yellow dashed lines represent hydrogen bonds. **E**: Structural model of TBX20 in complex with a palindromic DNA sequence containing a T-half site. Note: Green ribbon represents wild-type TBX20; orange ribbon represents TBX20-G193S. Involved residues are shown as spheres and colored by atom type (blue: nitrogen; white: hydrogen; red: oxygen; yellow: carbon)

Homology models of wild-type TBX20 (TBX20 WT) and the G193S variant were constructed and evaluated to investigate local structural alterations induced by the amino acid substitution. Residue 193 is located within an α-helical segment, flanked by an irregular coil and several β-strands (Fig. 1A). Structural comparison between the wild-type and variant models revealed that the loop region adjacent to Ser193 in the G193S variant shifted slightly outward, likely to accommodate the larger side chain of serine compared to glycine. This local rearrangement also influenced the conformation of adjacent structural elements (Fig. 1B).

In the wild-type model, Gly193 is positioned near Ala144, Thr192, and Leu197 and participates in a hydrogen bond network (Fig. 1C). In the G193S variant model, the substitution of glycine with serine disrupts these interactions. Specifically, the hydrogen bond between residue 193 and Leu197 is lost, and a new hydrogen bond is formed with Asp141. Additionally, Leu196 forms a new hydrogen bond in the variant model (Fig. 1D).

## Energetic and conformational effects of the G193S variant

Energy minimization calculations were performed to compare the potential energy states of the wild-type and variant TBX20 protein structures (Table 2). The results indicated that the G193S variant had a higher potential energy compared to the wild-type structure, suggesting reduced structural stability.

**Table 2:** TBX 20 energy table

Group	Potential Energy (kcal/mol)	Vander Waals Energy (kcal/mol)	Electrostatic Energy (kcal/mol)
Wild type	−11,142.7	−1194.8	−11,547.5
G193S	−10,872.1	−1252.5	−11,126.1
WT-DNA complex	−18,837.9	−1708.8	−20,854.9
G193S-DNA complex	−18,568.7	−1748.9	−20,651.9

The difference between energy after mutation and premutation energy is positive, indicating that energy increase and structural stability decrease; otherwise, stability increases

Both optimized structures were subsequently docked with a palindromic DNA sequence containing the T-half site (5′-AATTTCACACCTAGGTGTGTGAAATT-3′) to form complexes (Fig. 1E). The G193S variant remained in a high potential energy state within the complex, indicating decreased structural stability.

## Effect of T192T and G193S variants on TBX20 mRNA expression

**Fig. 2:**
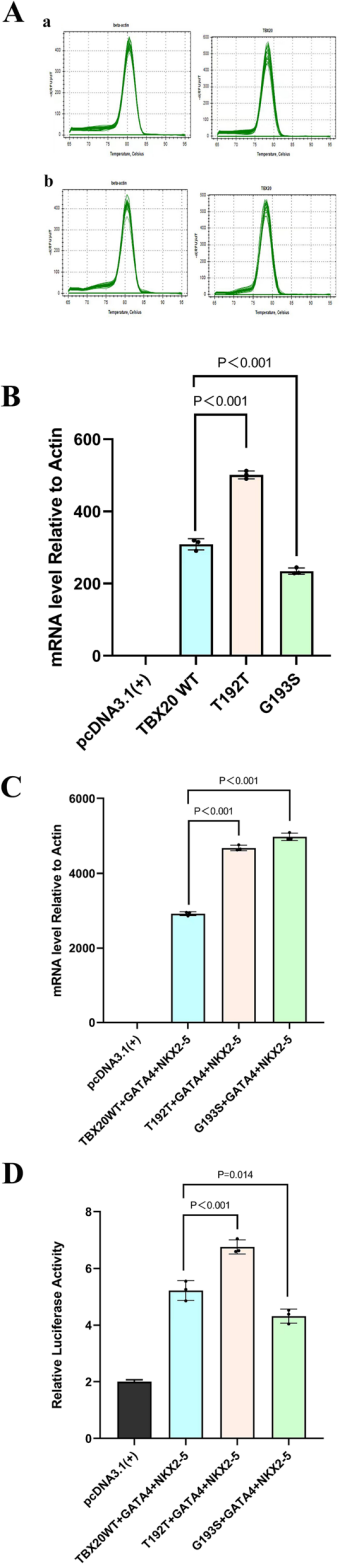
Functional analysis of TBX20 expression and activity. **A:** Melting curves from qRT-PCR showing amplification of TBX20 and *β-actin* in (**a**) solo TBX20 transfection and (**b**) co-transfection with GATA4 and NKX2-5. Note: The melting curve is the quality control pathway of the amplification reaction. No nonspecific amplification, primer-dimer formation, or contamination was observed. **B**: Relative TBX20 mRNA expression following transfection of wild-type and variant expression plasmids. TBX20-T192T expression was significantly higher than wild type, and TBX20-G193S expression was significantly lower (*p* < 0.001). **C**: Relative TBX20 mRNA expression following co-transfection of wild-type or variant TBX20 with GATA4 and NKX2-5. Expression levels of TBX20-T192T and TBX20-G193S were significantly higher than wild-type TBX20 (*p* < 0.001). **D**: Luciferase reporter assay showing *ANF* promoter activity under the influence of TBX20 variants with GATA4 and NKX2-5. TBX20-T192T significantly enhanced transcriptional activation compared to wild type, whereas TBX20-G193S significantly reduced activation (*p* < 0.05 for both comparisons)

Relative quantification of TBX20 mRNA expression levels was performed using RT-PCR, with β-actin as the internal reference. No contamination, primer dimer formation, or non-specific amplification was observed in the reactions (Fig. 2A).

Statistical analysis showed that mRNA expression of the TBX20-T192T variant was significantly higher than that of wild-type TBX20, whereas expression of the TBX20-G193S variant was lower than the wild type (Fig. 2B). This difference was statistically significant (*p* < 0.01; Table 3).

**Table 3:** Values and analysis of TBX20 WT and mutant mRNA expression during transfection alone (2-ΔΔCt analysis)

Sample name	beta-Actin(CT)	TBX20(CT)	2-ΔΔCt	2- ΔΔ Ct mean ± SD	*P*-value[1]
pcDNA3.1(+)	18.19	29.77	1.01911196	1.0004 ± 0.03638	
	18.22	29.80	1.023711977		
	18.23	29.90	0.958518097		
TBX20 WT	18.68	22.11	290.8372261	308.6572 ± 15.60434	4.0387E-10*
	18.90	22.21	315.2584503		
	19.00	22.29	319.8760715		
T192T	18.50	21.11	512.1750019	501.2771 ± 11.01823	1.6527E-8^△^ 8.3776E-12^*^
	18.52	21.16	501.513934		
	18.59	21.27	490.1423641		
G193S	18.37	22.04	244.5546658	234.3948 ± 8.81236	**0.000025**^△^ 3.6217E-9^*^
	18.37	22.14	229.8052289		
	18.51	22.28	228.8244814		

pcDNA3.1 (+): empty loaded plasmid transfected in COS-7 cells, used as blank control

TBX20 WT: For the wild-type TBX 20 expression plasmid transfected in COS-7 cells

T192T and G193S: mutant TBX 20 expression plasmids transfected in COS-7 cells

1 One-way ANOVA analysis was used

^*^The *P*-value obtained when compared with pcDNA3.1 (+)

△The *P*-values obtained in comparison with the TBX 20 WT

In co-transfection experiments with GATA4 and NKX2-5, both TBX20-T192T and TBX20-G193S variants exhibited significantly higher mRNA expression levels compared to wild-type TBX20 (Fig. 2C). These differences were also highly statistically significant (*p* < 0.001; Table 4).

**Table 4:** Expression of TBX20 gene mRNA after co-transfection with GATA4, NKX2-5

Sample name	beta-Actin(CT)	TBX20(CT)	2-ΔΔCt	2-ΔΔCt Means ± standard deviation	*P*-value[1]
pcDNA3.1(+)	19.18	30.35	1.003987907	1.0002 ± 0.02405	
	19.18	30.40	0.974471843		
	19.29	30.44	1.022120793		
TBX20WT + GATA4 + NKX2-5	19.16	18.81	2955.214803	2919.4303 ± 54.33072	1.8909E-11
	19.17	18.82	2946.163464		
	19.22	18.92	2856.912614		
T192T + GATA4 + NKX2-5	19.39	18.35	4764.408399	4681.2913 ± 72.99499	1.0526E-9^△^ 4.3496E-13^*^
	19.39	18.39	4651.854004		
	19.40	18.41	4627.611412		
G193S + GATA4 + NKX2-5	19.20	18.12	4917.639248	4978.6447 ± 100.93997	3.0459E-10^△^ 2.6585E-13^*^
	19.28	18.15	5095.156855		
	19.31	18.22	4923.137873		

pcDNA3.1 (+): empty loaded plasmid transfected in COS-7 cells, used as blank control

TBX20 WT + GATA 4 + NKX 2–5: COS-7 cells were transfected with TBX20 WT expression plasmid and co-transfected with GATA4 and NKX2-5 expression plasmids

T192T and G193S + GATA 4 + NKX 2–5: COS-7 cells were transfected with mutant TBX20 expression plasmid and co-transfected with GATA4 and NKX2-5 expression plasmids

1One-way ANOVA analysis was used

^*^The *P*-value obtained when compared with pcDNA3.1 (+)

△The *P*-value obtained in comparison with the TBX20 WT

In this study, the synonymous mutation (T192T) was predicted to have a low pathogenic potential by integrated model software. However, functional studies revealed that the mRNA expression level of the synonymous mutant TBX20-T192T was higher than that of wild-type TBX20, both when transfected alone and when co-transfected with the TBX20 expression vector. These findings suggest that the T192T mutation may influence TBX20 mRNA expression and transcriptional activity, potentially through altered DNA-binding affinity or regulatory mechanisms.

## Effect of TBX20 variants on *ANF* promoter activity

Luciferase reporter assay results demonstrated that activation of the *ANF* promoter by the TBX20-G193S variant was significantly reduced under the synergistic influence of GATA4 and NKX2-5 compared to wild-type TBX20 (Fig. 2D). This difference was statistically significant (*p* < 0.05; Table 5).

**Table 5:** Relative luciferase values and the statistical analysis

Group	Fiefly luciferase fluorescence values	Renilla luciferase fluorescence value	Relative fluorescence values	Mean	*P* values were analyzed by one-way ANOVA[1]
pcDNA 3.1(+)	9135	4703	1.942377206	2.000421887	-
	8821	4242	2.079443659		
	9341	4719	1.979444798		
TBX20WT + GATA4 + NKX2-5	14,462	2978	4.856279382	5.21963292	2.6856E-7
	12,904	2456	5.254071661		
	13,372	2410	5.548547718		
T192T + GATA4 + NKX2-5	19,494	2942	6.626104691	6.755539243	0.000006^*^0.000069^△^
	18,266	2770	6.594223827		
	18,419	2614	7.046289212		
G193S + GATA4 + NKX2-5	13,150	3255	4.039938556	4.31728809	0.000003^*^0.014^△^
	13,054	2885	4.524783362		
	12,420	2831	4.387142353		

pcDNA3.1 (+): empty loaded plasmid transfected in COS-7 cells, as blank control

TBX20 WT + GATA 4 + NKX 2–5: COS-7 cells were transfected with TBX 20 WT expression plasmid and cotransfected with GATA 4 and NKX 2–5

T192T and G193S + GATA 4 + NKX 2–5: COS-7 cells were transfected with mutant TBX 20 expression plasmid and co-transfected GATA 4 and NKX 2–5

[1]One-way ANOVA analysis was used

^*^The *P*-value obtained when compared with pcDNA3.1 (+)

^△^The *P*-values obtained in comparison with the TBX20 WT

In contrast, the TBX20-T192T variant showed enhanced activation of the *ANF* promoter relative to the wild-type, also with a statistically significant difference (*p* < 0.05; Table 5) (Fig. 2D). Moreover,a synonymous mutation c.576C > T (T192T) and a missense mutation c.577 G > A (G193S) were detected in the highly conserved T-box DNA-binding domain of TBX20 in five pediatric patients. Cross-species comparison of the G193S mutation in TBX20 showed that the affected amino acid is highly evolutionarily conserved. The protein structure model of TBX20 indicated that the mutation altered the relationship between the 193rd amino acid of the TBX20 gene and surrounding residues, and the altered protein conformation placed TBX20 and its complexes in a relatively unstable high-energy state, which may not be conducive to the binding of the TBX20 T-box DNA-binding domain and DNA sequences, thereby affecting cardiac development. Real-time quantitative PCR results showed that the mRNA expression level of the mutant TBX20-G193S was reduced. However, under the synergistic action of GATA4 and NKX2-5, the mRNA expression level of G193S increased. Yet, the increased expression did not strongly activate ANF as a mechanism. It may regulate other downstream target genes and change folding patterns, failing to activate or only partially activating downstream ANF, thus resulting in decreased regulation of ANF. This is consistent with our pathogenicity prediction of G193S, indicating that the G193S mutation can affect the mRNA expression of TBX20 and the influence of GATA4 and NKX2-5 on the mRNA expression of wild-type and mutant TBX20, which may be involved in the cardiac development mechanism.

In this study, the synonymous mutation (T192T) was found to have a low pathogenic possibility predicted by integrated model software. However, functional studies showed that whether transfected alone or co-transfected with the TBX20 expression vector, the mRNA expression level of the synonymous mutant TBX20-T192T was higher than that of wild-type TBX20. Under the synergistic action of GATA4 and NKX2-5, compared with wild-type TBX20, the transcriptional activation effect of TBX20-T192T on downstream target gene ANF was stronger. This study suggests that the synonymous mutation T192T can affect mRNA expression, enhance the regulation of ANF, and may be related to increased DNA binding affinity and enhanced transcriptional activity, which may affect cardiac development.

## Discussion

TBX20, a member of the T-box family of transcription factors, plays a critical role in cardiogenesis. Located on chromosome 7q14.2, the TBX20 gene consists of eight exons ranging in length from 289 to 888 nucleotides. These exons encode the T-box DNA-binding domain, which enables TBX20 protein to bind specific DNA sequences and regulate transcription. This domain is highly conserved across species, from *Drosophila* to humans.

During embryonic development, TBX20 activates transcription of multiple cardiac-expressed target genes, including *ANF*, *CX40*, and *CX45*, with *ANF* being among the most extensively studied. TBX20 expression in the embryonic heart is modulated through synergistic interaction with the cardiac transcription factors NKX2-5 and GATA4. Together, these factors influence the expression of genes critical for cardiac cell proliferation, differentiation, and compartmentalization during heart development [9, 10, 22]. Pathogenic variants in TBX20 may impair its interaction with other cardiac transcription factors, potentially disrupting this regulatory network and contributing to congenital heart defects.

Previous research has consistently reported associations between CHD and sequence variants in the TBX20 gene. These variants predominantly affect functionally important regions of the gene, including the T-box DNA-binding domain, transcriptional activation domain, and promoter region. For example, Posch et al. sequenced the TBX20 coding region in 170 individuals with atrial septal defect (ASD) type II and identified a novel variant (I121M) within a highly conserved residue of the DNA-binding domain. Functional assays demonstrated significantly increased transcriptional activity of the TBX20-I121M variant, particularly when co-expressed with the transcription factors GATA4, GATA5, and NKX2-5 [11].

Similarly, Huang et al. reported a nonsense variant (p.Lys274X) in an individual with tetralogy of Fallot. This variant introduces a premature stop codon within the exon encoding the DNA-binding domain, resulting in a truncated TBX20 protein lacking the C-terminal transcriptional activation and repression domains [23]. Functional assays showed that this variant was unable to activate the *ANF* promoter and exhibited reduced synergy with NKX2-5 and GATA4.

Qiao et al. identified a novel heterozygous variant (g.4932G > A) in the TBX20 promoter region in a patient with VSD, which significantly reduced promoter activity relative to the wild type [24]. Pan et al. reported a heterozygous variant (p.R143W) in a patient with double outlet right ventricle (DORV), with in vitro functional analysis revealing markedly reduced transcriptional activity compared to wild-type TBX20 [13].

Collectively, these studies expand the phenotypic spectrum associated with TBX20 variants and support their pathogenic potential in a subset of CHD cases. While CHD is multifactorial in origin, environmental influences such as maternal infections during pregnancy, medication exposure, nutritional deficiencies, and chemical exposures also contribute to its pathogenesis. Although efforts were made to exclude participants with known environmental risk factors, detailed exposure histories were not systematically collected. Therefore, while this study demonstrates an association between TBX20 variants and VSD, it cannot exclude the possibility that environmental factors may act synergistically in some affected individuals.

A synonymous variant, also referred to as a silent variant, is a nucleotide substitution that does not alter the amino acid sequence of the encoded protein. Traditionally, such variants were considered functionally neutral, with no effect on protein structure or function. However, recent studies have demonstrated that synonymous variants can influence a range of biological processes, including transcription factor binding, mRNA splicing, folding, degradation, and the initiation, efficiency, and fidelity of translation [25].

In 2020, Walsh et al. reported that synonymous variants can disrupt protein folding, thereby affecting cellular function [25]. More recently, in 2022, Shen et al. at the University of Michigan demonstrated that a substantial proportion of synonymous variants have deleterious effects, challenging the long-standing assumption that these changes are neutral or nearly neutral [26].

Despite initial predictions suggesting limited pathogenicity, the synonymous variant T192T identified in this study demonstrated functional effects on TBX20 expression. Specifically, mRNA levels of TBX20-T192T were higher than those of wild-type TBX20 in both solo transfection and co-transfection with GATA4 and NKX2-5. In the presence of these transcription factors, the T192T variant also exhibited enhanced transcriptional activation of the downstream target gene *ANF* compared to wild-type TBX20. These results suggest that the T192T variant may influence mRNA expression and transcriptional activity, potentially through increased DNA-binding affinity or altered transcriptional regulation, and may have implications for cardiac development.

This study has several limitations. First, detailed environmental exposure data were not systematically collected, which limits the ability to assess potential gene–environment interactions in VSD pathogenesis. Second, the sample size was relatively small and composed primarily of individuals of Han Chinese ethnicity from Shanxi Province, which may limit the generalizability of the findings. Future research should incorporate broader environmental assessments and include more ethnically diverse populations to validate and expand upon these results.

This study found that G193S mutation leads to a decrease in the transcriptional activation of downstream target gene ANF by TBX20, and affects its own mRNA expression. The analysis of the TBX20 protein structure prediction model shows that the G193S mutation to some extent affects its protein structure, which may be unfavorable for TBX20 to bind to specific DNA, and the change in its protein structure puts TBX20 and its complexes in a relatively unstable high potential state. The mutation of TBX20 gene may affect the regulation of downstream target gene ANF by TBX20 and other cardiac development related transcription factors, and further affect cardiac development, participating in the mechanism of CHD ventricular septal defect.

The co-occurrence of the synonymous T192T and missense G193S variants in the same sample suggests a complex interplay of regulatory effects on TBX20 expression. While T192T appears to enhance expression, G193S is associated with decreased expression and protein destabilization. This opposing dynamic underscores the importance of considering multiple variants in evaluating TBX20-related mechanisms in VSD.

Finally, the data suggest that TBX20 may be subject to autoregulatory feedback, potentially mediated by interactions with GATA4 and NKX2-5. The synergistic activation of downstream target genes by these transcription factors may extend to the regulation of TBX20 itself. Future studies should further investigate the molecular basis of this potential autoregulation and the compensatory roles of GATA4 and NKX2-5 in the context of TBX20 variants. Understanding these interactions may contribute to a more comprehensive view of VSD pathogenesis and identify novel therapeutic targets for CHD.

## Conclusion

This study demonstrated that the TBX20-G193S variant reduces *ANF* transcriptional activation and alters TBX20 mRNA expression, likely through disruption of protein structure and impairment of DNA-binding capacity, thereby potentially affecting cardiac development. In contrast, the synonymous T192T variant increases transcriptional activity and enhances *ANF* regulation, particularly in the presence of NKX2-5 and GATA4. These findings challenge the traditional assumption that synonymous variants are functionally neutral.

Future research should include the development of in vivo models, such as genetically modified mice carrying these variants, to elucidate the underlying mechanisms and to support improved strategies for the prevention and early intervention of CHD.

## Supplementary Information

Supplementary Material 1.

## Data Availability

No datasets were generated or analysed during the current study.

## Abbreviations

VSD: Ventricular septal defect
CHD: Congenital heart disease
PCR: Polymerase chain reaction
NCBI: National Center for Biotechnology Information
TBX20 WT: Wild-type TBX20
