# Supplementary material for: Structural and functional assessment of TBX20 gene variants in pediatric ventricular septal defect
Source: Hereditas. 2025 Aug 4;162:149. doi: 10.1186/s41065-025-00513-5 (PMC12323031; doi:10.1186/s41065-025-00513-5)
Supplement: Supplementary file 1 — Supplementary Material 1. [file 41065_2025_513_MOESM1_ESM.pdf]

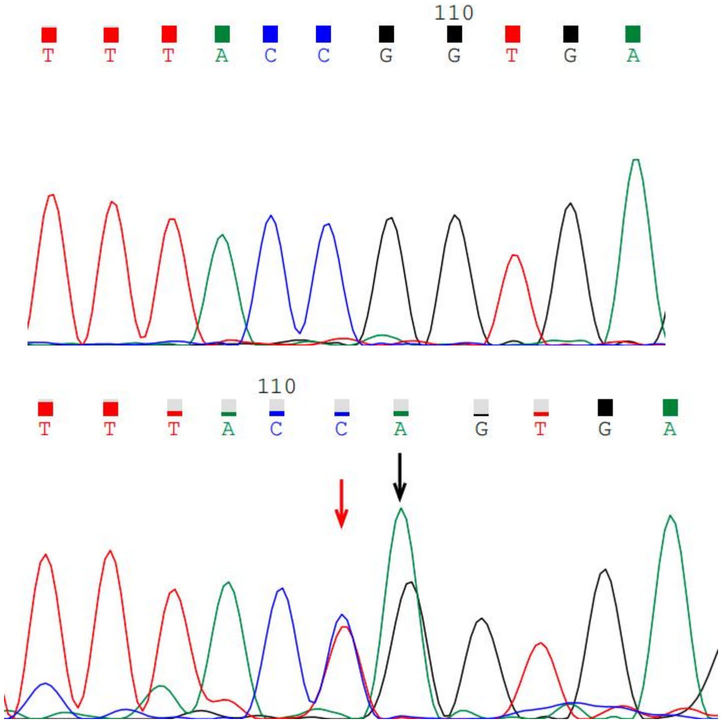

Supplementary Figure 1A: Sample 14

Synonymous mutation, T192T (c.576C/T , red arrow);

Missense mutation, G193S (c.577G/A , black arrow)

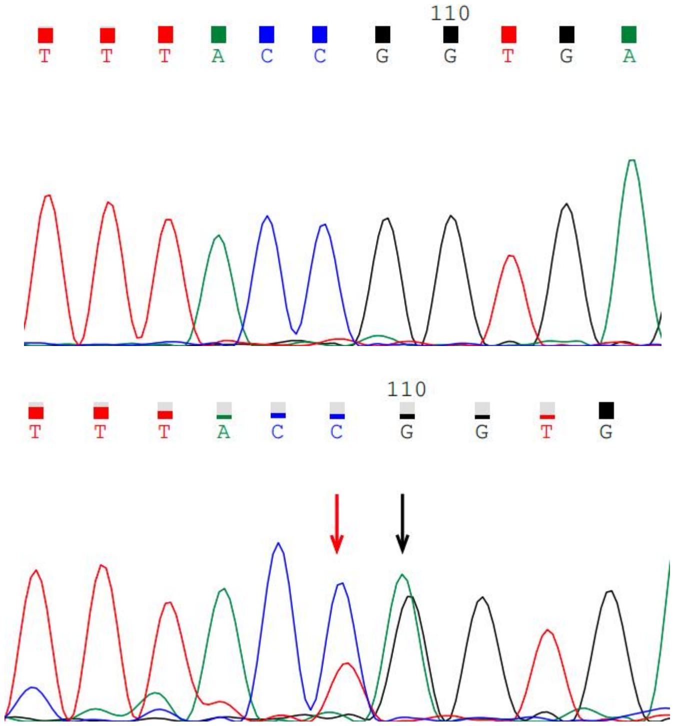

Supplementary Figure 1B: Sample 26

Synonymous mutation, T192T (c.576C/T, red arrow)

Missense mutation, G193S (c.577G/A , black arrow)

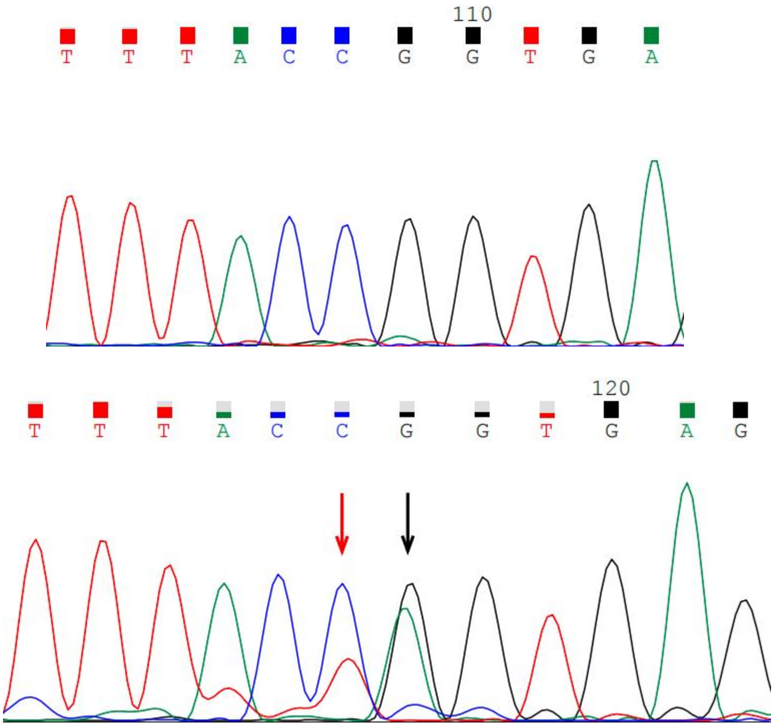

Supplementary Figure 1C: Sample 31

Synonymous mutation, T192T (c.576C/T , red arrow)

Missense mutation, G193S (c.577G/A , black arrow)

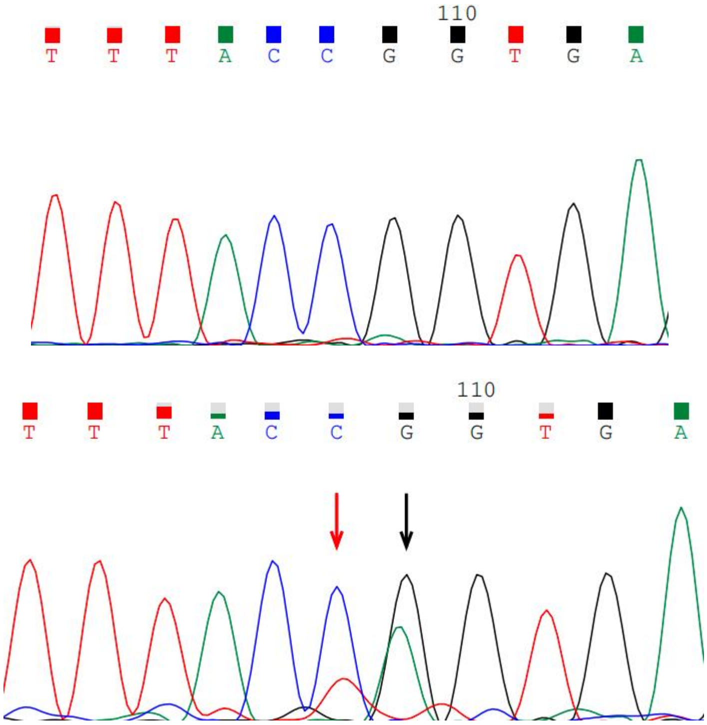

Supplementary Figure 1D: Sample 43

Synonymous mutation, T192T (c.576C/T, red arrow)

Missense mutation, G193S (c.577G/A , black arrow)

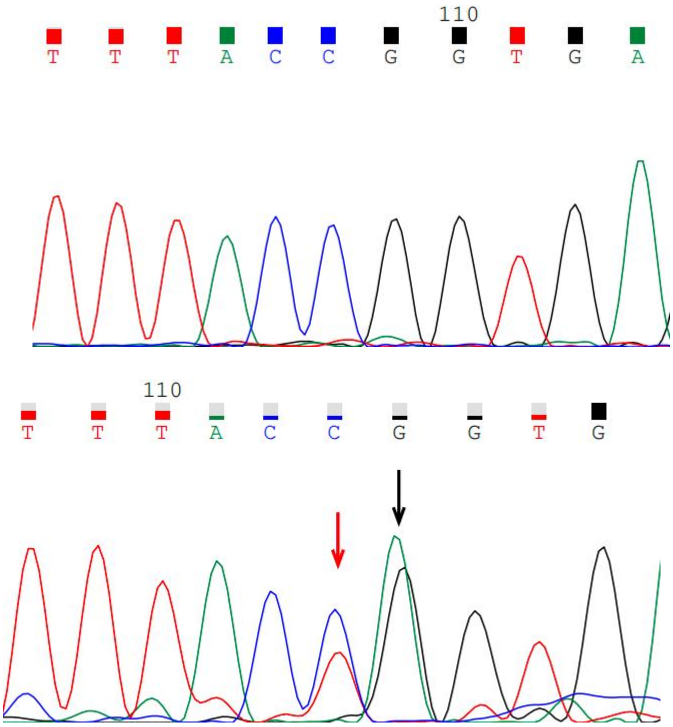

Supplementary Figure 1E: Sample 44

Synonymous mutation, T192T (c.576C/T , red arrow)

Missense mutation, G193S (c.577G/A , black arrow)

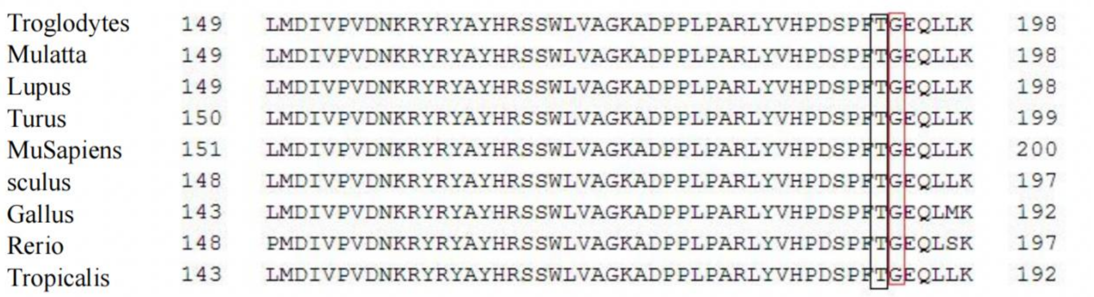

Supplementary Figure 2: The black box is the synonymous mutation (T192T), and the red box is the missense mutation (G193S) is highly conserved in human and animals

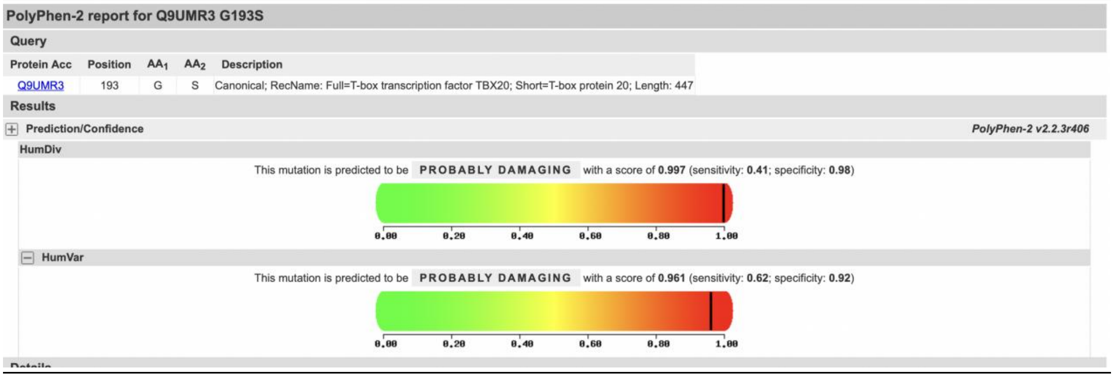

Supplementary Figure 3A: PolyPhen prediction of pathogenicity for G193S PROBABLY DAMAGING score is 0.98 (the closer to 1, the greater the deleteriousness)

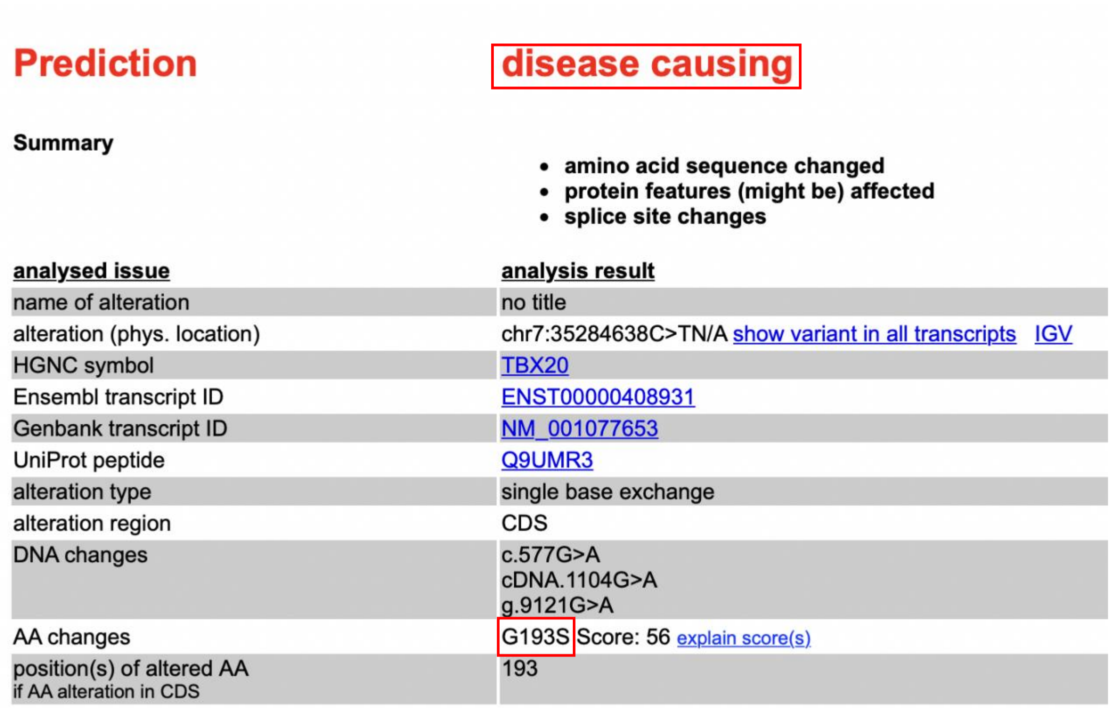

Prediction disease causing

Summary

- amino acid sequence changed
- protein features (might be) affected
- splice site changes

analysed issue	analysis result
name of alteration	no title
alteration (phys. location)	chr7:35284638C>TN/A show variant in all transcripts IGV
HGNC symbol	TBX20
Ensembl transcript ID	ENST00000408931
Genbank transcript ID	NM_001077653
UniProt peptide	Q9UMR3
alteration type	single base exchange
alteration region	CDS
DNA changes	c.577G>A<br>cDNA.1104G>A<br>g.9121G>A
AA changes	G193S Score: 56 explain score(s)
position(s) of altered AA<br>if AA alteration in CDS	193

Supplementary Figure 3B: Results of Mutation Taster pathogenicity prediction for G193S is Disease causing

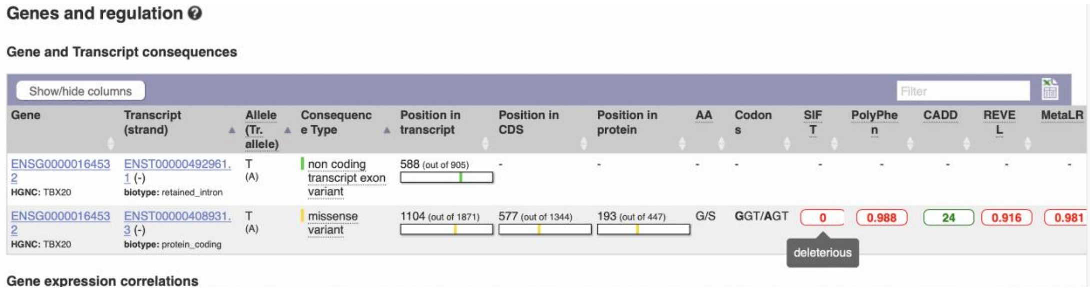

Genes and regulation

Gene and Transcript consequences

Gene	Transcript (strand)	Allele (Tr. allele)	Consequence Type	Position in transcript	Position in CDS	Position in protein	AA	Codons	SIFT	PolyPhen	CADD	REVEL	MetaLR
ENSG00000164532<br>HGNC: TBX20	ENST00000492961.1 (-)<br>biotype: retained_intron	T (A)	non coding transcript exon variant	588 (out of 905)	-	-	-	-	-	-	-	-	-
ENSG00000164532<br>HGNC: TBX20	ENST00000408931.3 (-)<br>biotype: protein_coding	T (A)	missense variant	1104 (out of 1871)	577 (out of 1344)	193 (out of 447)	G/S	GGT/AGT	0 (deleterious)	0.988	24	0.916	0.981

Gene expression correlations

Supplementary Figure 3C: Pathogenicity prediction results of G193S by SIFT is Deleterious

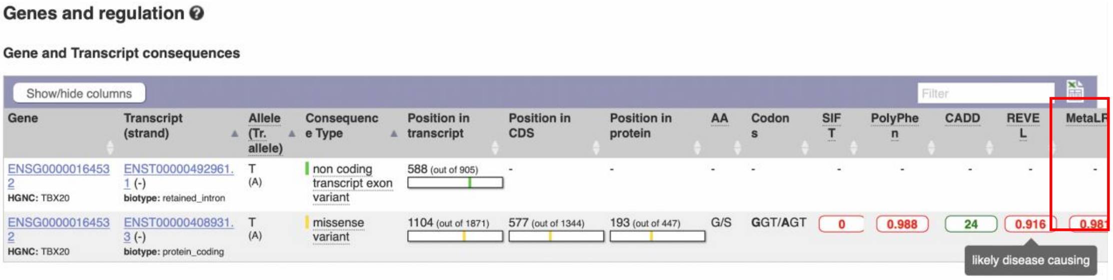

Supplementary Figure 3D: REVEL: The pathogenicity prediction results for G193S is likely desease causing, the score is 0.916 (the closer to 1, the greater the possibility of harm)

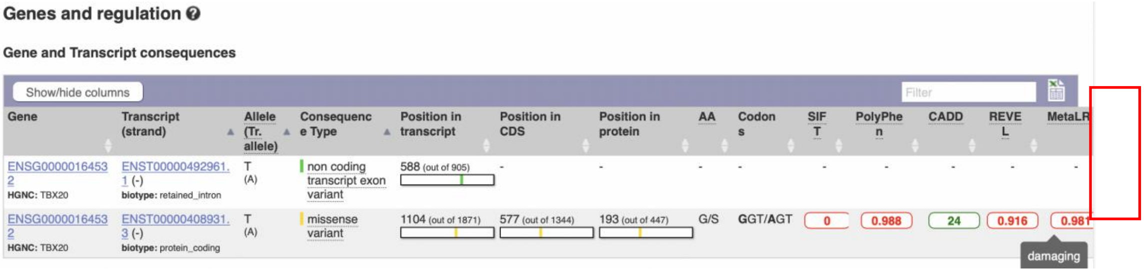

Supplementary Figure 3E: Results of MetalR pathogenicity prediction for G193S is damaging, the score is 0.981,

(the closer to 1, the more harmful)
